# Cross Talk: The Microbiota and Neurodevelopmental Disorders

**DOI:** 10.3389/fnins.2017.00490

**Published:** 2017-09-15

**Authors:** John R. Kelly, Chiara Minuto, John F. Cryan, Gerard Clarke, Timothy G. Dinan

**Affiliations:** ^1^Department of Psychiatry and Neurobehavioural Science, University College Cork Cork, Ireland; ^2^APC Microbiome Institute, University College Cork Cork, Ireland; ^3^Department of Anatomy and Neuroscience, University College Cork Cork, Ireland

**Keywords:** microbiota, microbiome, gut-brain axis, immune system, social cognition, autism, schizophrenia, psychobiotics

## Abstract

Humans evolved within a microbial ecosystem resulting in an interlinked physiology. The gut microbiota can signal to the brain via the immune system, the vagus nerve or other host-microbe interactions facilitated by gut hormones, regulation of tryptophan metabolism and microbial metabolites such as short chain fatty acids (SCFA), to influence brain development, function and behavior. Emerging evidence suggests that the gut microbiota may play a role in shaping cognitive networks encompassing emotional and social domains in neurodevelopmental disorders. Drawing upon pre-clinical and clinical evidence, we review the potential role of the gut microbiota in the origins and development of social and emotional domains related to Autism spectrum disorders (ASD) and schizophrenia. Small preliminary clinical studies have demonstrated gut microbiota alterations in both ASD and schizophrenia compared to healthy controls. However, we await the further development of mechanistic insights, together with large scale longitudinal clinical trials, that encompass a systems level dimensional approach, to investigate whether promising pre-clinical and initial clinical findings lead to clinical relevance.

## Introduction

From an evolutionary-based perspective, the host and its microbiome evolved as a cooperative unit (Rosenberg et al., 2007; Zilber-Rosenberg and Rosenberg, 2008; Martin et al., 2015; Douglas and Werren, 2016). All stages in the evolution of the human brain occurred within this microbial ecosystem (McFall-Ngai et al., 2013; Bordenstein and Theis, 2015). The predominant theory to account for the evolution of the enlargement of the human brain implicates social interaction. Brain areas such as the prefrontal cortex and the amygdala have undergone pronounced changes in the evolution of social mammals (Kolb et al., 2012; Janak and Tye, 2015). Brains of social species exhibit a set of features that need to integrate for group living to become advantageous, and the development of the complex neural circuitry underlying social and emotional cognition is of fundamental importance to neurodevelopmental disorders, such as ASD and schizophrenia (Adolphs, 2001; Lederbogen et al., 2011; Janak and Tye, 2015; Averbeck and Costa, 2017).

Neurodevelopment requires the intricate interplay of genetic expression, influenced by pre-and post-natal environmental events. Critical periods or “windows” of brain development exist, during which time neural circuits are particularly sensitive to, and require, the influence of appropriate environmental inputs, in order to develop properly. Human brain development begins in the third gestational week (Stiles and Jernigan, 2010) and at time of birth approximately 86 billion neurons (Azevedo et al., 2009) with up to 100 trillion connections are produced. These connections form simple circuits, and when reinforced through repeated use, under the influence of environmental cues, form more complex interconnected circuits, leading to complex networks (Bassett and Sporns, 2017). The developmental trajectory of social, emotional and cognitive brain domains occur in parallel, though social cognition may be linked to certain specific subnetworks (Dunbar, 2012; Sliwa and Freiwald, 2017).

The development of this neural circuitry requires precise regulation from molecular signaling pathways. Hormones, such as oxytocin (Kirsch et al., 2005), neurotransmitters, such as serotonin (Whitaker-Azmitia, 2001), and the immune system (Bilbo et al., 2012), all play pivotal roles in sculpting the neural circuitry underlying social cognition, emotion and behavior. Many of the brain regions involved and the molecular substrates subserving relevant functions are also responsive to microbiome-gut-brain axis signaling (Clarke et al., 2013; Semple et al., 2013; Montiel et al., 2014; Dinan et al., 2015; Erny et al., 2015; Buffington et al., 2016; Vuong and Hsiao, 2017) Figure 1.

**Figure 1:**
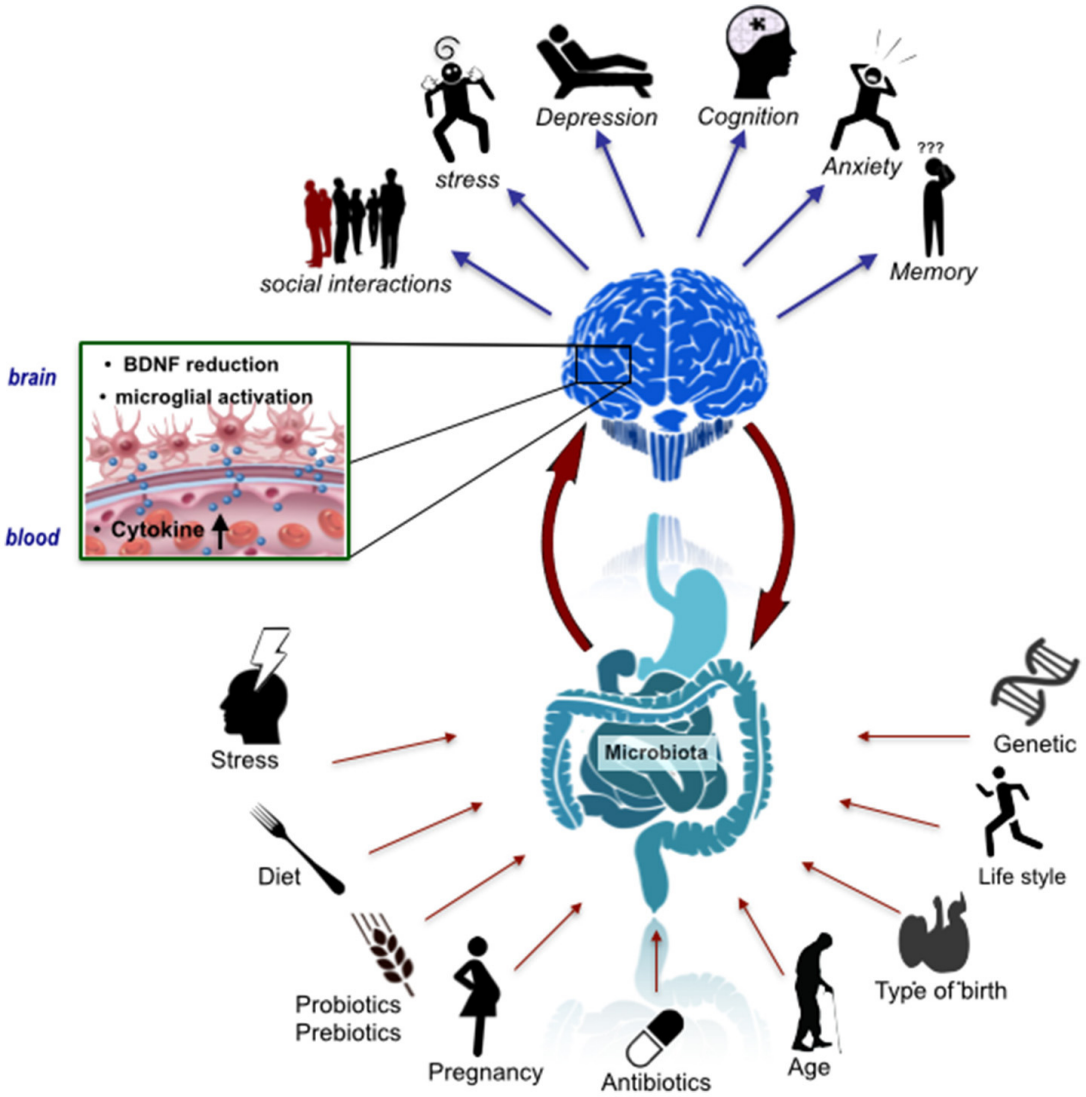
The microbiome-gut-brain axis in psychiatry. A number of factors have an influence on the assembly, composition and stability of the gut microbiota including mode of birth, lifestyle factors such as diet and exercise, and stress. These factors could thus impact signaling along the microbiome-gut-brain axis, which has been implicated in a variety of behavioral features relevant to schizophrenia and autism including anxiety and cognition. This impact may be underpinned by microbial regulation of the host immune system, CNS BDNF expression and microglial activation states.

The trajectory of early post-natal brain development overlaps with the acquisition and reorganization of the gut microbiota (Borre et al., 2014; Chu et al., 2017). The gut microbiota in the initial days of life is unstable and of low diversity (Arrieta et al., 2014). By age three, a stage by which verbal communication and Theory of Mind develops (the ability to infer and reason about the intentions, emotions and thoughts of others) (Grosse Wiesmann et al., 2017), the gut microbiota composition stabilizes into a pattern that more resembles an adult-like profile (Voreades et al., 2014). These social, cognitive and emotional domains, and their neurodevelopment, are compromised in neurodevelopmental disorders, such as ASD and schizophrenia. Deciphering the gut microbiota compositional trajectories and profiles, corresponding metabolic output and precise signaling pathways that play a pertinent role in molding the neural circuitry underlying the social-communicative domains of the brain, is one of the great challenges of modern neuroscience (Chen et al., 2013; Mayer et al., 2014a; Dinan and Cryan, 2017; Sherwin et al., 2017).

## Autism spectrum disorders (ASD)

ASD is a heterogeneous neurodevelopmental disorder, affecting approximately 1 in 68 children (Christensen et al., 2016). It is characterized by deficits in social communication, social interaction and restricted/repetitive behavioral patterns. The processing of emotional stimuli, be it language or facial expressions is impaired in individuals with ASD (Dalton et al., 2005; Preissler and Carey, 2005; Monk et al., 2010; Lartseva et al., 2014; Neuhaus et al., 2016; Wang and Adolphs, 2017). Therefore, deficits in social communication, together with emotional processing, and a lack of social interest in communication can result in language delay, and a proportion of children at the severe end of the spectrum will not develop language abilities (Landry and Loveland, 1988).

The heritability of ASD is estimated at between 64 and 91% (Tick et al., 2016), and genes that encode proteins for synaptic formation, microglial function, transcriptional regulation and chromatin-remodeling pathways are implicated (De Rubeis et al., 2014; Parikshak et al., 2016). New mutations contribute to the risk, and a recent large scale study, showed that approximately one third of spontaneous, non-inherited genetic mutations found in people with ASD were also found in the general population (Kosmicki et al., 2017). A recent study suggests that those ASD children with de novo mutations show relative strengths in verbal and language abilities, including a smaller discrepancy between non-verbal and verbal IQ and a greater likelihood of having achieved fluent language, relative to those with no identified genetic abnormalities (Bishop et al., 2017). Taken together, these genetic studies in ASD highlight the neurodiversity of the disorder (Baron-Cohen, 2017; Vorstman et al., 2017; Yuen et al., 2017).

The origins of ASD are likely to occur during the prenatal timeframe, a time window during which important connections are formed (Willsey et al., 2013). A study, using a high-resolution transcriptional atlas in primates, showed that many ASD-related genes are activated in new-born neurons during prenatal development, while schizophrenia related genes are activated from infancy through adulthood (Bakken et al., 2016). Maternal infections during pregnancy are associated with the development of neurodevelopmental disorders (Atladottir et al., 2010; Jiang et al., 2016; Careaga et al., 2017). Certain subtypes of ASD are associated with increased levels of maternal peripheral chemokines and cytokines during gestation (Goines et al., 2011; Jones et al., 2017; Graham et al., in press). Moreover, subgroups of children diagnosed with ASD have elevated levels of peripheral cytokines (Ashwood et al., 2011), and microglial activation in young adults with ASD has been demonstrated using positron emission tomography (PET) imaging (Suzuki et al., 2013). Progress is being made in diagnosing infants at high risk of developing ASD, by utilizing imaging techniques such as fMRI (Hazlett et al., 2017; Shen et al., 2017). A recent structural and diffusion MRI study of 3 year old infants diagnosed with Neurodevelopmental Disorders (32 ASD and 16 other developmental disorders, including intellectual disability and language disorder) reported an over-connectivity pattern in ASD in networks primarily involving the fronto-temporal nodes, known to be crucial for social-skill development (Conti et al., 2017).

Risk factors such as advanced parental age (Durkin et al., 2008; Sandin et al., 2016), low birth weight (Schendel and Bhasin, 2008) and multiple births (Croen et al., 2002) have been identified, while others such as mode of birth have been advanced. However, epidemiological data suggests that C-section mode of delivery, currently far in excess of WHO recommendations (WHO, 2015) and known to alter microbiome signatures (see below), is associated only with a slightly increased risk of ASD and that this may be due to familial confounds (Curran et al., 2014, 2015, 2016; O'Neill et al., 2015). Identifying additional modifiable environmental factors that play a causal role in ASD, particularly during the prenatal and early post-natal period, is of vital importance.

## Schizophrenia spectrum disorders (SSD)

Schizophrenia is a heterogeneous neurodevelopmental disorder, with a general population lifetime prevalence of approximately 0.87% (Perala et al., 2007), and an annual incidence of approximately 0.20/1,000/year (Messias et al., 2007). There is a slightly greater risk for males (Aleman et al., 2003) and psychotic symptoms usually manifest clinically during the adolescent period. This disorder can have a major detrimental impact on functioning, and is associated with a reduced life expectancy (Laursen et al., 2014; Schoenbaum et al., 2017; Strati et al., 2017) and a suicide rate of 5% (Hor and Taylor, 2010). schizophrenia is classically characterized by positive (delusions, hallucinations), negative (affective flattening, alogia, and avolition), and cognitive symptoms (Aleman et al., 1999; Kahn and Keefe, 2013; Schaefer et al., 2013). Social interaction and communication deficiencies, including disorganized speech, can be prominent, even early in the course of this disorder (Sullivan et al., 2003; Roche et al., 2016; Morgan et al., 2017).

Similar to ASD, the precise cause of schizophrenia is unknown. A complex and dynamic bidirectional interaction of genomic and environmental factors converge to shape the trajectory of schizophrenia (O'Tuathaigh et al., 2017). Prenatal and early post-natal environmental factors sensitize the vulnerable brain. Although psychotic symptoms usually manifest during the adolescent period, it has been established that schizophrenia is associated with poor premorbid functioning, cognitive impairment, and social deficits prior to the onset of psychotic symptoms (Schenkel and Silverstein, 2004). Indeed, previously considered distinct forms of psychopathology may in fact have characteristics in common, and exhibit age adjusted variations of common underlying dispositions (Casey et al., 2014; Hommer and Swedo, 2015).

The immune system is an important player in the pathophysiology of schizophrenia (Benros et al., 2012; Feigenson et al., 2014; Muller, 2014). At the genetic level, genes related to B-lymphocyte lineages involved in acquired immunity (CD19 and CD20 lines) and major histocompatibility complex locus have been linked to schizophrenia (Corvin and Morris, 2014; Schizophrenia Working Group of the Psychiatric Genomics Consortium., 2014). A recent translational study implicated excessive complement activity, particularly the role of C4 in mediating synapse elimination during post-natal development (Sekar et al., 2016). Neuro-immune signaling also changes during the adolescent period, regulating changes in synaptic pruning, neurite outgrowth, and neurotransmitter release via Blood Brain Barrier (BBB) dynamics and glial activity (Brenhouse and Schwarz, 2016). It is established that subgroups of patients with schizophrenia have elevated levels of peripheral cytokines, including subgroups of medication free first-episode psychosis individuals (Miller et al., 2011; Di Nicola et al., 2013; de Witte et al., 2014; Upthegrove et al., 2014). There is also some suggestion, though not well established, that schizophrenia is associated with altered intestinal (Severance et al., 2013) and blood brain barrier function (Pollak et al., 2017). This, taken together with altered microglial activation in psychosis patients (Bloomfield et al., 2016), highlights the key role of the immune system, in at least subgroups of individuals with psychosis (Al-Diwani et al., 2017).

Infections at different stages of brain development result in varying degrees of lifelong changes in behavior and cognition (Spencer and Meyer, 2017). Certain infections are known to increase the risk of schizophrenia (Meyer et al., 2009; Brown, 2012). A large epidemiological study (*n* = 1,015,447), showed that treatment with anti-infective agents (primarily driven by infections treated with antibiotics), were associated with an increased risk of schizophrenia by a hazard rate ratio of 1.37 (Kohler et al., 2017). However, an earlier study found an increased risk for mood and anxiety disorders for antibiotic exposure, but no change in risk for psychosis with any antibiotic group (Lurie et al., 2015). An infection with a robust link to psychosis is the protozoan *Toxoplasma gondii* (Torrey and Yolken, 2003; Severance et al., 2016b). A meta-analysis of 16 studies demonstrated increased *T. gondii* IgM levels in patients with acute psychosis (Monroe et al., 2015). The mechanism is not completely understood, but a putative role of attenuated CD8 T-cell response in *T. gondii* seropositive individuals has been suggested (Bhadra et al., 2013). It is known that *T. gondii* induces the production of a variety of cytokines by microglia, astrocytes, and neurons (Carruthers and Suzuki, 2007). Monocytes and dendritic cells are the most important candidates for the transport of *T. gondii* from the periphery to the immunologically privileged sites of the brain (Feustel et al., 2012).

Indeed, latent *T. gondii* infection is associated with an upregulation of cerebral complement factor C1q (Xiao et al., 2016). Furthermore, *T. gondii* infection can alter dopamine metabolism (Prandovszky et al., 2011) and latent *T. gondii* is associated with reduced psychomotor performance (Havlicek et al., 2001). More recently, *T. gondii* has been shown to lead to deficits in goal-directed behavior in healthy elderly individuals (Beste et al., 2014). Interestingly, acute *T. gondii* infection can affect the gut microbiota in mice (Molloy et al., 2013). Although only a minor subset of *T. gondii* seropositive individuals develop serious mental impairments, taken together, the example of *T. gondii*, suggests that microbial agents contribute to the vulnerability to the development of subgroups of schizophrenia (Yolken and Torrey, 2008). Although we have focussed on *T. gondii*, it is noteworthy that other infections, such as Human Herpesvirus 2, Borna Disease Virus Human Endogenous Retrovirus W, *Chlamydophila pneumoniae*, and *Chlamydophila psittaci* are also associated with the disorder (Arias et al., 2012). It remains an open question whether there is a common mechanism through which these microbes exert their influence, albeit, that one shared general feature in most examples cited is an intracellular life stage.

Interestingly, urbanicity, known to affect microbial diversity and impact the overall functionality of the gut microbiome (Mancabelli et al., 2017), is also a risk factor for the development of schizophrenia (Pedersen and Mortensen, 2001; Krabbendam and van Os, 2005; Peen et al., 2010; Vassos et al., 2012; Newbury et al., 2016). In healthy individuals, a negative correlation was found between early-life urbanicity and gray matter volume in the right dorsolateral prefrontal cortex in males and females, and in perigenual anterior cingulate cortex volumes, a key region for regulation of amygdala, in men only (Haddad et al., 2015). Using fMRI, city living was associated with increased amygdala activity (Lederbogen et al., 2011), known to be associated with schizophrenia (Aleman and Kahn, 2005; Rasetti et al., 2009). Stamper and colleagues postulate that differential exposure to microbes in the urban compared to the rural environment interact with differences in social stressors to alter social stress neural circuitry (Stamper et al., 2016).

## ASD and microbiota

GI symptoms are a common comorbidity in ASD (Molloy and Manning-Courtney, 2003; Buie et al., 2010; Berding and Donovan, 2016). However, the underlying mechanism is not fully known (Mayer et al., 2014b). The vast majority of human studies show that ASD is associated with altered microbial profiles (see Table 1). A systematic review of gut microbiota alterations in ASD, verified alterations in gut microbiota, but highlighted the heterogeneity of findings, and the limited quantity and quality of studies (Cao et al., 2013). Studies investigating ASD, the gut microbiota and SCFAs, showed significantly higher levels of *Desulfovibrio* species and *Bacteroides vulgatus* and higher levels of SCFA's in the stools of autistic children compared to controls (Finegold et al., 2010; Wang et al., 2012). *Clostridium Bolteae*, another species that is reported to be over-represented in the gut microbiota in ASD, and its capsular polysaccharide consisting of rhamnose and mannose units, has been proposed as a viable potential vaccine to reduce *C. bolteae* colonization of the intestinal tract in autistic patients (Pequegnat et al., 2013).

**Table 1:** Microbiota and ASD clinical studies.

**Design**	**Diagnosis, N, Age**	**Measures**	**Results**	**References**
Antibiotic—12-week trial of open label oral vancomycin	ASD, regressive-onset autism (*n* = 11) Age (43–84 months) No control group	Childhood Autism Rating Scale Developmental Profile II Coded, paired videotapes scored by a clinical psychologist blinded to treatment status	Behavioral improvement Improvement at follow-up (2–8 months)—not sustained	Sandler et al., 2000
FMT—18 weeks in total; 10 week open label and 8 week follow-up	ASD (*n* = 18) Age (7–16 years) Controls (*n* = 20) Age and Gender matched	Gastrointestinal Symptom Rating Scale Parent Global Impressions-III (PGI-II) Childhood Autism Rating Scale (CARS) Aberrant Behavior Checklist (ABC) Social Responsiveness Scale (SRS) Vineland Adaptive Behavior Scale II (VABS-II)	ASD-related behavior improved (PGI-II) (CARS) (SRS) (ABC)80% reduction of GI symptoms (persisted for 8 weeks post-FMT) *Bifidobacterium, Prevotella*, and *Desulfovibrio* increased post-FMT (persisted for 8 weeks post-FMT)	Kang et al., 2017
Cross-sectional	ASD, regressive-onset autism (*n* = 13) Controls (*n* = 8)	All ASD had GI symptoms (diarrhea and constipation) Gastric and small-bowel specimens (7 ASD, 4 controls) Limited dietary data: patients were on a gluten-free (GF), casein-free (CF) diet	ASD—more *Clostridial* species and non-spore-forming anaerobes and microaerophilic bacteria	Finegold et al., 2002
Cross-sectional	ASD (*n* = 20) Age (6.7 ± 2.7 years) 20 neurotypical children Age (8.3 ± 4.4 years)	Fecal samples Autism Diagnostics Interview—Revised (ADI-Revised) Autism Diagnostics Observation Schedule (ADOS) Autism Treatment Evaluation Checklist (ATEC) Pervasive Developmental Disorder Behavior Inventory (PDD-BI) Limited dietary data Most ASD had GI symptoms	ASD—less diverse gut microbial compositions with lower levels of *Prevotella, Coprococcus*, and unclassified *Veillonellaceae* Autistic symptoms, rather than the severity of GI symptoms, was associated with less diverse gut microbiota	Kang et al., 2013
Cross-sectional	ASD patients (*n* = 58) Age (3–16 years) Two control groups (*n* = 22); Non-autistic sibling group (*n* = 12) Age (2–10 years) Unrelated healthy group (*n* = 10) Age (3–12 years of age)	91.4% of ASD had GI Symptoms Limited dietary data; Most of the children were on GF/CF diets and many were taking probiotics/prebiotics/antibiotics	ASD—higher *Clostridium histolyticum* group compared to controls Non-autistic sibling group had an intermediate level of the *C. histolyticum* group – not significantly different from ASD or controls	Parracho et al., 2005
Cross-sectional	ASD (*n* = 23) Age (123 ± 9 months) Controls (*n* = 31) Age (136 ± 9 months)	SCFAs Dietary intake of macro-nutrients	ASD—fecal acetic, butyric, isobutyric, valeric, and isovaleric acid were all significantly higher compared with controls	Wang et al., 2012
Cross-sectional	ASD (*n* = 40) Age (11.1 ± 6.8 years) Neurotypical controls (*n* = 40) Age (9.2 ± 7.9 years)	Childhood Autism Rating Scale (CARS) Autism Diagnostic Observation Schedule and Autism Behavior Checklist Constipation defined according to Rome III criteria All subjects of in this study were on a Mediterranean-based diet, and no antibiotics, probiotics, or prebiotics taken in the 3 months prior to the sample collection	ASD—increase in the *Firmicutes/Bacteroidetes* ratio due to a reduction of the *Bacteroidetes* relative abundance ASD—at the genus level—decrease in *Alistipes, Bilophila, Dialister, Parabacteroides*, and *Veillonella*, while *Collinsella, Corynebacterium, Dorea*, and *Lactobacillus* were significantly increased Constipated ASD—high levels of bacterial taxa belonging to *Escherichia/Shigella* and *Clostridium cluster XVIII* ASD—fungal genus Candida increased	Strati et al., 2017
Cross-sectional	ASD (*n* = 23, without GI symptoms) ASD (*n* = 28, with GI symptoms) Age range (2–12 years) Neurotypical siblings (*n* = 53) Age range (2–12 years)	Childhood Autism Rating Scale (CARS) Limited dietary data; Probiotics not excluded	No significant differences in microbiota	Gondalia et al., 2012
Cross-sectional	ASD (*n* = 15, with GI symptoms) Age (4.5 ± 1.3 years) Controls (*n* = 7, with GI symptoms) Age (4.0 ± 1.1 years)	Autism Diagnostic Interview-Revised (ADI-R) Intestinal biopsies	ASD with GI symptoms had a decrease in disaccharidases and hexose transporters, and decreases in *Bacteroidetes*, increase in *Firmicutes/Bacteroidetes* ratio, and increase in *Betaproteobacteria* compared with controls with GI symptoms	Williams et al., 2011
Cross-sectional	ASD (*n* = 15) Control (*n* = 8)	Diet not recorded	ASD—elevated levels of *Clostridium boltea* and *Clostridium group I and XI*	Song et al., 2004
Cross-sectional	ASD (*n* = 58, GI symptoms) Age (6.91 ± 3.4 years) Controls (*n* = 39) Age (7.7 ± 4.4 years)	GI symptoms (assessed by the six-item GI Severity Index (6-GSI) questionnaire) Autism Treatment Evaluation Checklist (ATEC) Diet not recorded, ASD on probiotics	ASD—decreased fecal SCFAs, acetate, proprionate, and valerate ASD—lower levels of *Bifidobacterium* and higher levels of *Lactobacillus* GI symptoms were strongly correlated with the severity of autism	Adams et al., 2011
Meta-analysis of 15 cross-sectional studies			11 studies (*n* = 562) reported significant gut microbiota differences between ASD children and controls, particularly in the *Firmicutes, Bacteroidetes* and *Proteobacteria* phyla Substantial heterogeneity in methodology and the often contradictory results of different studies—not possible to pool the results into a meta-analysis	Cao et al., 2013
Cross-sectional	ASD children (*n* = 23) Age (123 ± 9 months) Controls (*n* = 31); Typically developing siblings (*n* = 22) Community controls (*n* = 9) Age (136 ± 9 months)	Macronutrient intake determined from dietary records kept by caregivers, did not differ significantly between study groups	ASD—elevated fecal acetic, butyric, isobutyric, valeric, isovaleric, and caproic acids, ammonia	Wang et al., 2012
Cross-sectional	ASD (*n* = 33, varying GI symptoms) Controls (*n* = 15); 7 sibling controls 8 non-sibling controls Age (all ASD and controls between 2 and 13 years)	No diet	*Bacteroidetes* was found at high levels in the severely autistic group *Firmicutes* were more predominant in the control group Smaller, but significant, differences also in the *Actinobacterium* and *Proteobacterium* phyla *Desulfovibrio* species and *Bacteroides vulgatus* present in significantly higher numbers in stools of severely autistic children than in controls	Finegold et al., 2010
Probiotic Intervention—“Children Dophilus” oral capsule containing 3 strains of *Lactobacillus* (60%), 2 strains of *Bifidumbacteria* (25%) and one strain of *Streptococcus* (15%), times a day for 4 months	ASD (*n* = 10) Age (2–9 years) Siblings (*n* = 9) Age (5–17 years) Controls (*n* = 10) Age (2–11 years)	Autism Diagnostic Interview (ADI) Childhood Autism Rating Scale (CARS)	ASD—decrease of the *Bacteroidetes/Firmicutes* ratio and elevation of the amount of *Lactobacillus* *Desulfovibrio* decreased postprobiotic *Desulfovibrio* spp. associated with the severity of autism (ADI) restricted/repetitive behavior subscale score Probiotic significantly decreased fecal TNFα levels in ASD No correlation between plasma levels of oxytocin, testosterone, DHEA-S and fecal microbiota	Tomova et al., 2015
Cross-sectional	Healthy children (*n* = 77) Age (18–27 months)	Early Childhood Behavior Questionnaire (ECBQ) (18 dimensions of temperament, three composite scales: Negative Affectivity, Surgency/Extraversion, Effortful Control)	Greater surgency/extraversion was associated greater phylogenetic diversity Boys only—subscales loading on this composite scale were associated with differences in phylogenetic diversity, the Shannon Diversity index (SDI), beta diversity, and differences in abundances of *Dialister, Rikenellaceae, Ruminococcaceae*, and *Parabacteroides* Higher effortful control was associated with a lower SDI score and differences in both beta diversity and Rikenellaceae were observed in relation to Fear Associations between temperament and dietary patterns were observed	Christian et al., 2015
Cross-sectional	ASD (*n* = 17) Asperger's syndrome (*n* = 6) Mean age (123 ± 9 months) 22 typically developing siblings Age (144 ± 12 months) Community controls (*n* = 9) Age (114 ± 15 months)	Functional gastrointestinal disorder (FGID) questionnaire Antibiotics/probiotics not excluded Some on Gluten- and casein-free diet	ASD—Low Relative Abundances of the Mucolytic Bacterium and *Akkermansia muciniphila* and *Bifidobacterium spp*. in Feces	Wang et al., 2011
Cross-sectional	ASD (*n* = 23, 3 without siblings) 22 typically developing siblings Age (144 ± 12 months) Community controls (*n* = 9) Age (114 ± 15 months)	No diet	ASD—*Sutterella* spp. elevated in feces relative to controls and *Ruminococcus torques* higher in the children with ASD with a reported functional gastrointestinal disorder than those without such a disorder	Wang et al., 2013
Cross-sectional	ASD (*n* = 10) Pervasive Developmental Disorder Not Otherwise Specified (PDD-NOS) (*n* = 10) Healthy controls (HC) siblings (*n* = 10) Age (all 4–10 years)	Autism Diagnostic Interview-Revised (ADI-R) Autistic Diagnostic Observation Schedule (ADOS) Childhood Autism Rating Scale (CARS) Diet not recorded No antibiotics, probiotics and prebiotics for at least 1 month before sampling	ASD—highest microbial diversity *Faecalibacterium* and *Ruminococcus* were present at the highest level in fecal samples of PDD-NOS and HC children. *Caloramator, Sarcina* and *Clostridium* genera were the highest in ASD children Except for *Eubacterium siraeum*, the lowest level of *Eubacteriaceae* was found in fecal samples of ASD *Bifidobacterium* species decreased in AD—Compared to HC children Altered levels of free amino acids and volatile organic compounds of fecal samples in ASD and PDD-NOS	De Angelis et al., 2013
Cross-sectional	ASD probands (*n* = 66) Neurotypical (NT) siblings (*n* = 37) Age (7–14 years)	Parent-completed ROME III questionnaire for pediatric Functional gastrointestinal disorders (FGIDs) Child Behavior Check List (CBCL) Targeted quantitative polymerase chain reaction (qPCR) assays were conducted on selected taxa implicated in ASD, including *Sutterella* spp., *Bacteroidetes* spp., and *Prevotella* spp.	No significant difference in macronutrient intake between ASD and NT siblings There was no significant difference in ASD severity scores between ASD children with and without FGID No significant difference in diversity or overall microbial composition was detected between ASD children with NT siblings	Son et al., 2015

Most studies conducted in ASD are non-interventional, and many do not adequately record detailed dietary information or medication use. Indeed, it is well established that ASD is highly associated with atypical eating patterns (Cermak et al., 2010). The interventional studies are few, and of small sample size. An open labeled trial (*n* = 11), with no control group, using the poorly absorbed oral antibiotic, vancomycin for 12 weeks, reportedly resulted in a short-term improvement in ASD related behavioral symptomatology in a group of children with regressive-onset autism (Sandler et al., 2000). Follow-up which occurred between 2 and 8 months, showed that the improvement was not sustained. More recently, a small (*n* = 18) open label study of Fecal Microbiota Transfer (FMT) in children with ASD reported an improvement in both GI symptoms and behavioral symptoms after 8 weeks (Kang et al., 2017). In this study, the abundance of *Bifidobacterium, Prevotella*, and *Desulfovibrio* increased following the 8 weeks of FMT treatment (see Table 1). Interestingly, in a cross sectional study, in healthy prepubertal children (*n* = 65) dietary fiber was associated with a better performance on a task measuring attentional inhibition (Khan et al., 2015). Moreover, a study investigating microbial composition at 1 year of age showed that a higher alpha diversity was associated with lower scores on the Mullen scale, the visual reception scale, and the expressive language scale at 2 years of age (Carlson et al., in press).

## Schizophrenia spectrum disorder and the microbiota

As discussed below, data from pre-clinical studies indicate that certain domains related to schizophrenia, such as social cognition, are under the partial influence of the gut microbiota (Dinan et al., 2014). However, pre-clinical models have many limitations and translating promising pre-clinical findings into discernible clinical benefits for patients can be challenging, particularly for complex disorders such as schizophrenia. Moreover, it is important to highlight that there are considerable interpersonal differences in the gut microbiota profiles of healthy individuals (Backhed et al., 2012; Falony et al., 2016; Zhernakova et al., 2016). Consequently, there are multiple possible configurations for a healthy gut microbiota and it is also likely that some stable configurations are associated with disorders (Relman, 2012). It is important also to appreciate that the functional output of multiple microbiota configurations may in fact be equivalent, given that concepts of redundancy and pleiotropy can also be applied to specific microbial members of the overall consortium (Falony et al., 2016).

Despite the significant challenges, several pilot clinical studies investigating the microbiome in schizophrenia have emerged (see Table 2). A recent study investigating the gut microbiota in schizophrenia was conducted in First Episode Psychosis (FEP) patients (*n* = 28) compared to healthy controls (*n* = 16) (Schwarz et al., in press). There were five significant differences between the groups at the family level; *Lactobacillaceae, Halothiobacillaceae, Brucellaceae*, and *Micrococcineae* were increased, whereas *Veillonellaceae* were decreased in FEP patients. At the genus level, *Lactobacillus, Tropheryma, Halothiobacillus, Saccharophagus, Ochrobactrum, Deferribacter*, and *Halorubrum* were increased, and *Anabaena, Nitrosospira*, and *Gallionella* were decreased in FEP. *Lactobacillus* group bacterial numbers correlated positively with severity of psychotic symptoms measured using the Brief Psychiatric Rating Scale, and negatively with global assessment of functioning (GAF) scale. A subgroup analysis of those classified as less physically active, confirmed significant increases in *Lactobacillaceae* and significant decreases in *Veillonellaceae* in FEP. It is noteworthy that the vast majority of FEP patients were prescribed antipsychotic medication, which can impact gut microbiota composition (Davey et al., 2012, 2013; Bahra et al., 2015; Bahr et al., 2015). A small study (*n* = 32) of the oropharyngeal microbiome in schizophrenia also showed an increased abundance of *Lactobacillus* in schizophrenia patients, in addition to, *Bifidobacterium* and *Ascomycota*, compared to healthy controls (Castro-Nallar et al., 2015). Another study of the oral pharynx of 41 individuals with schizophrenia and 33 controls demonstrated that one bacteriophage genome *Lactobacillus phage phiadh*, was significantly more abundant in schizophrenia patients than in controls after adjustment for multiple comparisons and demographic covariates (Yolken et al., 2015).

**Table 2:** Microbiota and clinical schizophrenia (SCZ) studies.

**Design**	**Diagnosis, N, Years**	**Measures**	**Results**	**References**
Cross-sectional	Schizophrenia (*n* = 16) Years (34.7 ± 4.8) Controls (*n* = 16) Years (34.3 ± 10.1) Differences in smoking and BMI between groups	Shotgun metagenomic analysis of the oropharyngeal microbiome	SCZ—higher proportions of *Firmicutes, Ascomycota, Bifidobacterium* and *Lactobacilli* (largest effect was observed in *Lactobacillus gasseri*) SCZ—increase *Candida* and *Eubacterium* and reduction of *Neisseria, Haemophilus* and *Capnocytophaga* SCZ—increased number of metabolic pathways related to metabolite transport systems including siderophores, glutamate and vitamin B12 Carbohydrate and lipid pathways and energy metabolism were abundant in controls	Castro-Nallar et al., 2015
Cross-sectional	Schizophrenia (*n* = 41) Years (39.2 ± 9.9) Controls (*n* = 33) Years (30.9 ± 8.8) Differences in smoking, BMI and age	Metagenomic analysis to characterize bacteriophage genomes in oral pharynx	SCZ—increased *Lactobacillus phage phiadh* (controlling for age, gender, race, socioeconomic status, or smoking)	Yolken et al., 2015
Two case-control cohorts (*n* = 947)	Schizophrenia (*n* = 261), including; First-episode schizophrenia (*n* = 139, 78 antipsychotic naïve) Years (37.71 ± 13.69) Bipolar (*n* = 270) Years (34.08 ± 13.15) Controls (*n* = 277) Years (32.02 ± 11.31)	Repeatable Battery for the Assessment of Neuropsychological Status (RBANS)	No differences in *C. albicans* exposures were found until diagnostic groups stratified by sex SCZ—in males, *C. albicans* seropositivity conferred increased odds (OR 2.04–9.53) for a SCZ diagnosis SCZ—in females, *C. albicans* seropositivity conferred increased odds (OR 1.12) for lower cognitive scores on RBANS with significant decreases on memory modules *C. albicans* IgG levels were not impacted by antipsychotic medications Gastrointestinal (GI) disturbances were associated with elevated *C. albicans* in males with SCZ and females with bipolar	Severance et al., 2016a
14 week double-blind, placebo controlled *.Lactobacillus rhamnosus strain GG* and *Bifidobacterium animalis* subsp. *lactis Bb12* (10^9^ cfu)	Schizophrenia (*n* = 56) Probiotic (*n* = 30) Placebo (*n* = 26) Years (44.66 + 11.4)	Biweekly Positive and Negative Syndrome Scale (PANSS) Self-reported—bowel score (scale of 1–4)	SCZ—in males—reduced *C. albicans* antibodies *S. cerevisiae* were not altered Trends toward improvement in positive psychiatric symptoms in males treated with probiotics who were seronegative for *C. albicans*	Severance et al., 2017
Lactobacillus rhamnosus strain GG and Bifidobacterium animalis subsp. lactis Bb12 (10^9^ cfu) 14 week double-blind, placebo controlled	Schizophrenia (*n* = 65) 33 probiotic Years (44.4 ± 11.0) 32 placebo Years (48.1 ± 9.4) All on antipsychotic medication	Positive and Negative Syndrome Scale (PANSS) every 2 weeks Self-reported—bowel score (scale of 1–4)	No significant differences in the PANSS Probiotic group—significantly less likely to develop severe bowel difficulty	Dickerson et al., 2014
Longitudinal (12 months)	First Episode Psychosis (FEP) (*n* = 28) Years (25.9 ± 5.5) Most on antipsychotics Healthy controls (*n* = 16) Years (27.8 ± 6.0)	Brief Psychiatric Rating Scale (BPRS) Global assessment of functioning (GAF) scale Diet adapted from “Health Behavior and Health among the Finnish Adult Population” survey	FEP—at family level; *Lactobacillaceae, Halothiobacillaceae, Brucellaceae and Micrococcineae* were increased whereas *Veillonellaceae* were decreased FEP—at genus level; *Lactobacillus, Tropheryma, Halothiobacillus, Saccharophagus, Ochrobactrum, Deferribacter* and *Halorubrum* were increased, and *Anabaena, Nitrosospira* and *Gallionella* were decreased *Lactobacillus* group bacterial numbers correlated positively with severity of psychotic symptoms measured using the BPRS and negatively with GAF scale	Schwarz et al., in press

Studies investigating the fungal composition of the human gut—the Mycobiome—are also emerging (Suhr and Hallen-Adams, 2015). A case-control cohort study that included 261 individuals with schizophrenia, 270 with bipolar disorder, and 277 non-psychiatric controls, found no differences in *C. albicans* exposure when analyzed at the group level. However, when stratified by sex, there was a reported increase in the odds for schizophrenia in males (Severance et al., 2016a). The same group conducted a randomized, double-blind, placebo-controlled, probiotic trial over a 14-week period, and showed that probiotic treatment significantly reduced *C. albicans* antibodies in males only, and a trend toward improvement in positive psychiatric symptoms in seronegative males (Severance et al., 2017). Both groups were prescribed antipsychotic medication, but antipsychotic regimes were not different between probiotic and placebo groups.

## Communication pathways of brain-gut-microbiota axis

The human body contains as many bacterial cells as human cells (Sender and Fuchs, 2016), the majority of which reside in the gut, with bacterial concentrations ranging from 10^1^ to 10^3^ cells per gram in the upper intestines to 10^11^–10^12^ bacteria per gram in the colon (O'Hara and Shanahan, 2006; Derrien and van Hylckama Vlieg, 2015). With over 1,000 species and 7,000 strains the microbiota is an ecosystem dominated by bacteria, mainly strict anaerobes, but also includes viruses and bacteriophages, protozoa, archaea and fungi (Lankelma et al., 2015). In terms of bacterial phyla found in the gut, *Firmicutes* (species such as *Lactobacillus, Clostridium, Enterococcus*) and *Bacteroidetes* (species such as *Bacteroides*) account for the majority (Dethlefsen et al., 2007), though the other phyla such as *Actinobacteria (Bifidobacteria), Proteobacteria (Escherichia coli), Fusobacteria, Verrucomicrobia*, and *Cyanobacteria* are also present in relatively low abundance (Eckburg et al., 2005; Qin et al., 2010; Lankelma et al., 2015).

Although the functional significance of the gut microbiota has yet to be fully determined (Franzosa et al., 2014; Cani, 2017), it is clear that an intricate and interlinked symbiotic relationship exists between host and microbe (Ley et al., 2008), and there are a number of bidirectional signaling pathways by which the gut microbiota, acting via the brain-gut axis, can impact the brain. A key signaling pathway involves modulation of the immune system (Erny et al., 2015), though other pathways include the hypothalamic-pituitary-adrenal (HPA) axis (Sudo et al., 2004; Mudd et al., 2017), tryptophan metabolism (O'Mahony et al., 2015), the production of bacterial metabolites, such as SCFA (Tan et al., 2014) and via the vagus nerve (Bravo et al., 2011). Although much progress has been made, the precise signaling pathways mediating the influence of microbial products derived from gut microbiota on the brain remain largely unknown. Epigenetic factors may also play a role (Dalton et al., 2014; Stilling et al., 2014a,b; Thaiss et al., 2016). Recently, a novel signaling pathway has been advanced, that involves bacterial peptidoglycan (PGN) derived from the commensal gut microbiota (Arentsen et al., 2017). PGN was shown to translocate into the brain to activate specific pattern-recognition receptors (PRRs) of the innate immune system, and this could occur in both physiological and pathological conditions (Arentsen et al., 2017).

In addition, pre-clinical evidence from germ-free (GF) mice suggests that the microbiota can modulate the Blood Brain Barrier (BBB). Exposure of GF adult mice to the fecal microbiota from pathogen-free donors decreased BBB permeability (Braniste et al., 2014). Moreover, monocolonization of the intestine of GF adult mice with SCFA-producing bacterial strains normalized BBB permeability, whilst sodium butyrate was associated with increased expression of the tight junction protein occludin in the frontal cortex and hippocampus (Braniste et al., 2014). Together with a study that showed antibiotic-induced gut dysbiosis reduced the expression of tight junction proteins (claudin and occludin) mRNA in the hippocampus, and increased the expression of tight junction protein 1 and occludin mRNA in the amygdala (Frohlich et al., 2016), suggests that the BBB may be partially modulated by changes in the gut microbiota.

## Microbiota and the immune system

A bidirectional communication system exists between the immune system and the CNS. Neuroimmune signaling during the prenatal or early post-natal developmental stages can have long lasting effects on the brain, and is an important determinant of cognitive function and emotional behavior (Dantzer et al., 2008; Bilbo et al., 2012; Filiano et al., 2017; Freytag et al., 2017). Peripheral cytokine signaling can modulate astrocytes, microglia and neurons in the CNS (Kohman and Rhodes, 2013). This occurs through leaky regions in the BBB such as circumventricular organs, active transport through transport molecules, activation of cells lining the cerebral vasculature (endothelial cells and perivascular macrophages), binding to cytokine receptors associated with the vagus nerve, stimulating the HPA axis at the anterior pituitary or hypothalamus and recruitment of activated cells such as monocytes/macrophages from the periphery to the brain (Haroon et al., 2012). In addition, functional lymphatic vessels lining the dural sinuses have been discovered, which serve as a route by which immune cells can communicate with the CNS (Louveau et al., 2015). Consequently, peripheral cytokines can modulate neurogenesis, synapse formation and plasticity (Hodes et al., 2015). It is established that cytokines can impact cognition and mood (Dowlati et al., 2010; Udina et al., 2012; Valkanova et al., 2013; Khandaker et al., 2014). Brain regions affected by administration of inflammatory stimuli include the basal ganglia and the dorsal anterior cingulate cortex (dACC), part of the limbic system, involved in cognitive and emotional processing (Harrison et al., 2009; Slavich et al., 2010; Capuron et al., 2012; Felger and Miller, 2012; Felger et al., 2013; Miller et al., 2013).

A critical function of the gut microbiota is to prime the development of the neuroimmune system (Round and Mazmanian, 2009; Olszak et al., 2012; Chistiakov et al., 2014; Francino, 2014). Alterations in the gut microbiota signature early in life can predispose to immune disorders (Penders et al., 2007; Fujimura et al., 2016) and the luminal surface of the gut is a key interface in this process (O'Hara and Shanahan, 2006). Indeed, the hygiene hypothesis first proposed in the late 1980's (Strachan, 1989; Patel and Gruchalla, 2017) and reconceptualized as the “old friends hypothesis” (Rook et al., 2003; Williamson et al., 2015) proposes that encountering less microbial biodiversity may contribute to the increase in chronic inflammatory disorders (Klerman and Weissman, 1989; Guarner et al., 2006; Rook and Lowry, 2008; Turnbaugh et al., 2009; Hidaka, 2012; Rook et al., 2013, 2014; Kostic et al., 2015; Stein et al., 2016). An intriguing strategy of “reintroducing” old friends has been suggested by a pre-clinical study using heat-killed *Mycobacterium vaccae*, an immunoregulatory environmental microorganism. Mice given this vaccine exhibited reduced subordinate, flight, and avoiding behavioral responses to a dominant aggressor in a murine model of chronic psychosocial stress when tested 1–2 weeks following the final immunization, compared to the control group (Reber et al., 2016). Depletion of regulatory T cells negated the protective effects of immunization with *M. vaccae* on anxiety-like or fear behaviors.

## Mechanistic influences of microbiota on brain function and development

### Toll-like receptors (TLRs)

Structural components of bacteria interact with the immune system via TLRs (McCusker and Kelley, 2013). Different TLRs recognize specific bacterial structures, for example; TLR2 recognizes structures from Gram positive bacteria whereas TLR4 mediates responses to structures such as lipopolysaccharide (LPS) primarily from Gram negative bacteria (Marteau and Shanahan, 2003). In the CNS, neurons and glial cells can express various TLRs (Bsibsi et al., 2002; Kielian, 2006; Trudler et al., 2010). Activation of TLRs trigger the induction of pro and anti-inflammatory cytokines (Takeda and Akira, 2005) and, as mentioned above, there are a number of routes by which peripheral cytokines can impact the brain (Haroon et al., 2012; Miller et al., 2013; Louveau et al., 2015). Dysregulation of this process, or excessive TLR activation, can result in chronic inflammatory and over-exuberant repair responses. Consequently, TLRs may serve as molecular communication channels between gut microbiota alterations and immune system homeostasis (Rogier et al., 2015). Indeed, TLR2 and TLR4 knockout mice showed subtle impairments in behavior and cognitive functions (Park et al., 2015; Too et al., 2016). A clinical study in subjects diagnosed with psychotic disorders showed specific alterations in TLR agonist-mediated cytokine release compared to healthy controls (McKernan et al., 2011), and more recently it has been shown that abnormal expression of TLRs can be modulated by antipsychotics (Kéri et al., 2017). Moreover, in post-mortem prefrontal cortex samples from subjects diagnosed with psychosis, alterations in TLR4 have been shown, which were dependent on antipsychotic treatment status at time of death (García-Bueno et al., 2016).

### Microbiota and microglia

Microglia, central to the inflammatory process (Facci et al., 2014) are emerging as playing key roles in brain development, plasticity and cognition (Tay et al., 2017). These phagocytic innate immune cells account for approximately 10% of cells in the brain (Prinz et al., 2014), contribute to the plasticity of neural circuits by modulating synaptic architecture and function (Graeber and Streit, 2010) and can be modulated by glutamatergic and GABAergic neurotransmission (Fontainhas et al., 2011). Pre-clinical studies have shown that acute stress results in microglia activation and increased levels of proinflammatory cytokines in areas such as the hippocampus (Frank et al., 2007) and hypothalamus (Blandino et al., 2009; Sugama et al., 2011). Most studies show increases in activated microglia in response to chronic stress (Tynan et al., 2010; Hinwood et al., 2011, 2012; Bollinger et al., 2016).

Preliminary changes in the microenvironment of the microglia may result in a susceptibility to a secondary inflammatory stimulus (Perry and Holmes, 2014). This concept of microglia priming may be of relevance to neurodevelopmental disorders, such as ASD and schizophrenia, which often require multiple environmental “hits” (Feigenson et al., 2014; Fenn et al., 2014). In an environmental two-hit rodent model in which the first experimental manipulation targeted pregnant dams, and the second manipulation was given to the resulting offspring, exposure to prenatal immune challenge and peripubertal stress synergistically induced pathological effects on adult behavioral functions and neurochemistry (Giovanoli et al., 2013, 2015). Thus, early-life stress may prime microglia, leading to a potentiated response to subsequent stress (Calcia et al., 2016).

In human studies, microglial dysregulation has been demonstrated in several psychiatric disorders. In medication free depressed patients, microglial activation has been demonstrated in the prefrontal cortex, ACC, and insula, using translocator protein density measured by distribution volume in a PET study positron emission tomography (PET) study (Setiawan et al., 2015). Using a different tracer, (11)[C]PBR28, subjects at high risk of psychosis, and those with schizophrenia also showed evidence of altered microglial activation compared to healthy controls (van Berckel et al., 2008; Bloomfield et al., 2016). However, not all studies are consistent and no clear consensus exists (Holmes et al., 2016; Narendran and Frankle, 2016; Collste et al., 2017; Notter and Meyer, 2017).

The gut microbiota, emerging as an important neuroimmunomodulator (Foster, 2016; Rea et al., 2016), is also involved in the maturation and activation of microglia (Cryan and Dinan, 2015; Erny et al., 2015). Interestingly, GF mice display underdeveloped and immature microglia in the cortex, corpus callosum, hippocampus, olfactory bulb, and cerebellum (Erny et al., 2015). There was an upregulation of microglia transcription and survival factors, and downregulation of cell activation genes and genes for type 1 IFN receptor signaling compared with those isolated from conventionally colonized control mice. These defects were partially restored by recolonization with a complex microbiota, and SCFAs reversed the defective microglia in the absence of complex microbiota (Erny et al., 2015). Collectively, these studies suggest that subtle alterations in gut microbiota acquisition and development, by regulating neuro-inflammatory processes, may act as additional vulnerability factors that predispose to neurodevelopmental disorders such as ASD and schizophrenia.

## Microbiota and neurochemistry

At the cellular level, brain development and function requires a complex and coordinated birth, migration and differentiation of both neurons and glia, followed by synaptic integration and neural circuit formation. Both ASD and schizophrenia are associated with dysregulation of synaptic function and structure (McGlashan and Hoffman, 2000; Faludi and Mirnics, 2011; Spooren et al., 2012; Habela et al., 2016). The gut microbiota plays a role in developmental programming of the brain, specifically, synapse maturation and synaptogenesis (Diaz Heijtz et al., 2011) Figure 2. Synaptophysin, a marker of synaptogenesis, and PSD 95, a marker of excitatory synapse maturation, were decreased in the striatum in GF animals compared to specific-pathogen-free (SPF) animals. This suggests that the gut microbiota may programme certain brain circuits when colonized by maternal microbiota. However, the authors point out that exposure to gut microbiota metabolites during embryogenesis may also be a possible mechanism. Interestingly, reduced levels of synaptophysin have been demonstrated in the cerebral cortex of post-mortem samples from schizophrenia subjects (Hu et al., 2015).

**Figure 2:**
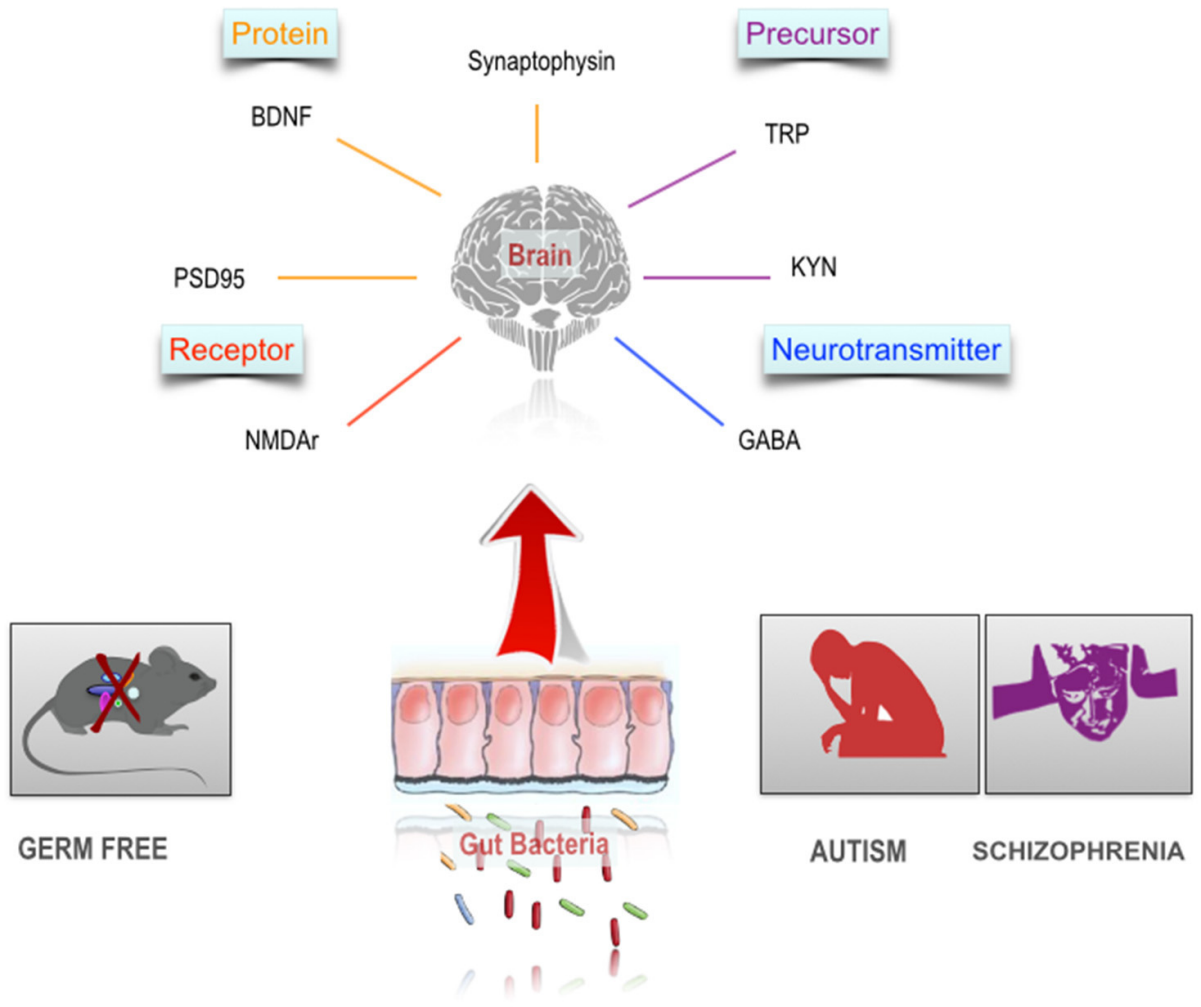
The gut microbiome and the neurobiology of schizophrenia and autism. Autism and schizophrenia are associated with a number of alterations in the CNS including altered availability of neuroactive precursors. Studies in germ free animals indicate a substantial overlap between these neurobiological characteristics and the scope of influence of the gut microbiome in the CNS.

### Brain-derived neurotrophic factor (BDNF)

A key regulator of synaptic plasticity and neurogenesis in the brain, throughout life, is the neurotrophin, BDNF (Monteggia et al., 2004). Given the role of BDNF in the regulation of synaptic strengthening and pruning, maintaining appropriate levels of BDNF and other neurotrophins, especially during critical neurodevelopmental windows is vital for both ASD and schizophrenia (Nieto et al., 2013). Meta-analysis showed reduced blood levels in both medication naïve and medicated adult individuals diagnosed with schizophrenia (Green et al., 2011). Conversely, children with ASD have increased levels of blood BDNF (Qin et al., 2016; Saghazadeh and Rezaei, 2017). In GF rodents, levels of BDNF were reduced in the cortex and hippocampus in GF mice (Sudo et al., 2004). In a study by Clarke et al. this finding was replicated, but in male mice only (Clarke et al., 2013). However, not all studies are consistent; Neufeld et al. (2011) confirmed a decreased level of anxiety like behavior in GF animals, but found an increase in BDNF mRNA in the hippocampus in female mice. Prebiotics can alter BDNF levels (Savignac et al., 2013) and increase BDNF gene expression in the hippocampus (Burokas et al., in press). Collectively, these pre-clinical studies suggest that certain neurotransmitters and neuromodulators of relevance to the pathophysiology of ASD and schizophrenia are under the influence of the gut microbiota Figure 2.

### γ-aminobutyric acid (GABA) and glutamate

At the neurotransmitter level, several signaling pathways have been shown to be dysfunctional in ASD and schizophrenia. Glutamatergic and GABAergic dysfunction and the consequences on excitatory to inhibitory cortical activity is one hypothesis to account for the similarities in the social and cognitive disturbances in ASD and schizophrenia (Canitano and Pallagrosi, 2017). GABA is an important inhibitory neurotransmitter in the brain, and GABA dysfunction has been implicated in ASD and schizophrenia (Schmidt and Mirnics, 2015). Although not a central source, it is interesting to note that certain bacteria can produce neuroactive metabolites (Wikoff et al., 2009; Lyte, 2011, 2013), for example specific strains of *Lactobacillus* and *Bifidobacteria* can produce GABA by metabolizing dietary glutamate (Barrett et al., 2012). Indeed, *L. rhamnosus* (JB-1) was shown to reduce anxiety and depression related behavior in mice and increase GABA receptor levels in the hippocampus (Bravo et al., 2011). Interestingly, in vagotomized mice, these effects were not found, further supporting the concept that the vagus nerve is an important neural signaling pathway between the microbiota and brain. A pre-clinical magnetic resonance spectroscopy study adds further evidence to support the concept that oral *L. rhamnosus* can increase central GABA levels (Janik et al., 2016). In a recent study, prebiotics, fructo-oligosaccharide (FOS) and galacto-oligosaccharide (GOS), increased GABA-B1 and GABA-B2 receptor gene expression in the hippocampus (Burokas et al., in press).

The glutamate hypothesis of schizophrenia, has suggested that hypofunction of signaling through NMDA receptors (NMDARs) plays a causal role in schizophrenia (Gonzalez-Burgos and Lewis, 2012). The glutamatergic system appears to contribute to certain cognitive deficits in schizophrenia (Thomas et al., 2017). Similarly, glutamatergic dysfunction has been implicated in ASD (Rojas, 2014). In GF mice Neufeld and colleagues demonstrated a decrease in the NMDAR subunit NR2B mRNA expression in the amygdala (Neufeld et al., 2011) Figure 2. Although a review of post-mortem studies of subjects with schizophrenia found consistent evidence of morphological alterations of dendrites of glutamatergic neurons in the cerebral cortex, there were no consistent alterations of mRNA expression of glutamate receptors (Hu et al., 2015).

### Serotonin

Serotonin (5-HT) has a wide range of physiological functions, and is involved in the modulation of anxiety, conditioned fear, stress responses, reward, and social behavior (Lucki, 1998; Dayan and Huys, 2008; Asan et al., 2013). A meta-analysis of post-mortem studies found an elevation in prefrontal 5-HT1A receptors and a reduction in prefrontal 5-HT2A receptors in schizophrenia (Selvaraj et al., 2014). Serotonin, and its pre-cursor tryptophan, are critical signaling molecules in the brain-gut-microbiota axis (O'Mahony et al., 2015). In GF mice decreased 5-HT1A in hippocampus has been shown (Neufeld et al., 2011). In the gastrointestinal tract (GI), 5-HT plays an important role in secretion, sensing and signaling (Mawe and Hoffman, 2013). The largest reserve of 5-HT is located in enterochromaffin cells (Berger et al., 2009). Emerging evidence also suggests that the serotonergic system may be under the influence of gut microbiota, especially, but not limited to, periods prior to the emergence of a stable adult-like gut microbiota (Desbonnet et al., 2008; El Aidy et al., 2012; Clarke et al., 2013). A metabolomics study demonstrated that the gut microbiota has a significant impact on blood metabolites and showed an almost three-fold increase in plasma serotonin levels when GF mice are colonized by gut microbiota (Wikoff et al., 2009). The gut microbiota itself is also an important regulator of 5-HT synthesis and secretion. For example, colonic tryptophan hydroxylase 1 (Tph1) mRNA and protein were increased in humanized GF and conventionally raised mice. Bacterial metabolites have also been demonstrated to influence Tph1 transcription in a human enterochromaffin cell model (Reigstad et al., 2015). Others have demonstrated that distinct microbial metabolites produced by spore forming bacteria increase colonic and blood 5-HT in chromaffin cell cultures (Yano et al., 2015).

### Kynurenine

The regulation of circulating tryptophan availability, and the distribution and subsequent kynurenine pathway metabolism, in the periphery and CNS, is tightly regulated during all stages of life (Ruddick et al., 2006; Badawy, 2017). The enzyme indoleamine 2,3-dioxygenase (IDO) found in macrophages and microglia cells is the first and rate limiting step in the kynurenine pathway of tryptophan catabolism. The expression of tryptophan-2,3-dioxygenase (TDO) can be induced by circulating glucocorticoids (O'Connor et al., 2009) and has been reported to be regulated by the gut microbiota during colonization (El Aidy et al., 2014). Under normal physiological conditions, approximately 99% of tryptophan is metabolized to kynurenine in the liver by TDO. However, proinflammatory cytokines such as IFN-γ, CRP, IL-1, IL-6, and TNF-α can induce IDO resulting in the metabolism of tryptophan along the kynurenine pathway (Schwarcz et al., 2012). Kynurenine, tryptophan and 3-hydroxykynurenine (3-HK) can cross the BBB and tryptophan's conversion to kynurenine and 3-HK in the peripheral circulation can therefore contribute to CNS levels (Schwarcz et al., 2012; Myint and Kim, 2014). In the brain, kynurenine metabolism occurs in all cells, though the two kynurenine pathway branches are physically segregated into distinct cell types. Astrocytes contain kynurenine aminotransferases (KATs), not kynurenine 3-monooxygenase (KMO) and therefore cannot produce 3-hydroxykynurenine (3-HK) from Kynurenine (Guidetti et al., 2007). The end result of the metabolic pathway in astrocytes is the neuroprotective Kynurenic acid (KYNA) (Gramsbergen et al., 1997), whereas, in microglia, it is the neurotoxic metabolite quinolinic acid (Alberati-Giani et al., 1996).

As mentioned above, regulation of the kynurenine pathway is important throughout life, but especially during sensitive periods of early neurodevelopment. KYNA is an NMDA and alpha7 nicotinic (α7nACh) receptor antagonist, both important in modulating brain development (Myint and Kim, 2014). Administration of kynurenine, starting during embryogenesis, reduced the expression of α7nACh receptor and mGluR2 expression, and induced deficits in prefrontal cortex mediated cognition in adult rats (Pershing et al., 2015). Indeed, prenatal, but not adolescent, kynurenine treatment caused significant impairments in hippocampal-mediated behavioral tasks (Pocivavsek et al., 2014). Combining perinatal choline-supplementation, with embryonic kynurenine manipulation, to potentially increase activation of α7nACh receptors during development, can attenuate cognitive impairments in adult rat offspring (Notarangelo and Pocivavsek, 2017). Furthermore, prenatal kynurenine induces age-dependent changes in NMDA receptor expression (NR2A, NR1) (Pershing et al., 2016). This study also showed that juvenile rats that were given kynurenine performed better in a trace fear conditioning task, whereas the adults showed deficits. Prenatal inhibition of kynurenine pathway, using the kynurenine-3-monoxygenase inhibitor (Ro61-8048), results in altered synaptic transmission and protein expression in the brains of adult offspring (Forrest et al., 2013; Khalil et al., 2014; Pisar et al., 2014), and also changes hippocampal plasticity (Forrest et al., 2015). Using a kynurenine 3-monooxygenase knockout mouse model (Kmo−/−), which increased brain KYNA levels, showed impairments in contextual memory, social behavior, and increased anxiety-like behavior (Erhardt et al., in press). Interestingly, administering D-amphetamine to Kmo−/− mice showed potentiated horizontal activity in the open field paradigm.

In schizophrenia, increased KYNA levels in CSF, including in drug naïve patients (Nilsson et al., 2005), and in post-mortem brain samples have been shown (Erhardt et al., 2001; Plitman et al., 2017). In a clinical study, patients with schizophrenia (*n* = 64) were more intolerant to a psychological stress challenge than healthy controls, and while salivary KYNA levels increased significantly between baseline and 20 min following the stressor in both patients and controls, patients who were unable to tolerate the stressful tasks showed significantly higher levels of KYNA than patients who tolerated the psychological stressor or healthy controls (Chiappelli et al., 2014). A recent pre-clinical study showed that restraint stress in pregnant mice caused significant elevations of KYNA levels in the maternal plasma, placenta, and fetal brain (Notarangelo and Schwarcz, 2017). Furthermore, the kynurenine/tryptophan ratio was significantly higher in patients diagnosed with psychotic disorder (Barry et al., 2009). Collectively, these pre-clinical and clinical studies highlight the importance of the kynurenine pathway during neurodevelopment, and there is a growing appreciation that integrating these important insights with the emerging importance of microbial regulation of this pathway will be an important research objective (Kennedy et al., 2017).

### Zinc signaling

The essential micronutrient Zinc plays an important role in immune function and GI development and function (Kau et al., 2011; Vela et al., 2015). Multiple independent factors affect Zinc status, including diet, prenatal and early life stress, immune system dysregulation, and impaired GI function (Vela et al., 2015). Zinc deficiency, particularly during the prenatal phase, has been proposed as an environmental risk factor for ASD. Indeed, in rats, acute Zinc deficiency can result in hyperactivity and over-responsivity, whereas prenatal deficiency can impair vocalizations and social behavior (Grabrucker et al., 2014). It has been suggested that the post-synaptic protein Shank3, which is localized at synapses in the brain and is associated with neuro-developmental disorders such as ASD and schizophrenia, is an important component of zinc-sensitive signaling system that regulates excitatory synaptic transmission, and may lead to cognitive and behavioral abnormalities in infants with ASD (Grabrucker et al., 2014; Arons and Lee, 2016). In clinical studies, Zinc deficiency has been reported in infants with ASD (Yorbik et al., 2004; Yasuda et al., 2011; Li et al., 2014). However, studies investigating Zinc levels in schizophrenia have yielded inconsistent results (Cai et al., 2015). The impact of micronutrient imbalances on the gut microbiota are beginning to emerge (Hibberd et al., 2017). In a study using chicks, Zinc deficiency induced gut microbiota alterations and decreased species richness and diversity (Reed et al., 2015). Excess dietary Zinc significantly altered the gut microbiota and in turn reduced the threshold of antibiotics needed to confer susceptibility to *C. difficile* infection in mice (Zackular et al., 2016).

### Epigenetic influences

Dietary factors can result in epigenetic alterations that lead to disease susceptibility (Jirtle and Skinner, 2007). It has been established that prenatal malnutrition increases the risk of schizophrenia (Susser and Lin, 1992; St Clair et al., 2005; Xu et al., 2009). Furthermore, it has been suggested that the microbiota is an important mediator of gene–environment interactions (Stilling et al., 2014b). SCFAs (butyrate, acetate and propionate) are neurohormonal signaling molecules produced by certain classes of bacteria such as *Bacteroides, Bifidobacterium, Propionibacterium, Eubacterium, Lactobacillus, Clostridium, Roseburia, and Prevotella* (Macfarlane and Macfarlane, 2012). SCFAs are transported by monocarboxylate transporters, which notably are expressed at the BBB (Steele, 1986; Vijay and Morris, 2014). A pre-clinical imaging study demonstrated that microbiota-derived acetate can cross the BBB where it can subsequently alter hypothalamic gene expression (Frost et al., 2014). Butyrate has been shown to be associated with increased expression of the tight junction protein occludin in the frontal cortex and hippocampus (Braniste et al., 2014). Butyrate, which acts as a potent inhibitor of Histone deacetylase (HDAC), is also a ligand for a subset of G protein-coupled receptors (Bourassa et al., 2016). It is clear that supra-physiological levels do have marked behavioral consequences (MacFabe et al., 2007; Macfabe, 2012; Thomas et al., 2012). However, the ability of physiological levels of SCFAs to substantially effect behavior via central mechanism are likely to be subtle, though cumulative chronic delivery may produce long-lasting stable effects on gene expression.

### Microbiota and social behavior and cognition

Neuronal activity in the amygdala is altered in GF mice (Stilling et al., 2015). In these mice, expression of immediate early response genes such as Fos, Fosb, Egr2, or Nr4a1 were increased in the amygdala, in conjunction with increased signaling of the transcription factor CREB (Stilling et al., 2015). Differential expression and recoding of several genes involved in fundamental brain processes ranging from neuronal plasticity, metabolism, neurotransmission and morphology were identified and a significant downregulation was noted for immune system-related genes (Stilling et al., 2015). In addition to an altered transcriptional profile in the amygdala, GF mice have recently been shown to exhibit reduced freezing behavior during a cued memory retention test, while colonized GF mice were behaviorally comparable to conventionally raised mice during the retention test (Hoban et al., 2017). Furthermore, adult GF mice have distinct dendritic morphological changes in the amygdala and hippocampus (Luczynski et al., 2016) and myelination of the prefrontal cortex has also been shown to be under the influence of the gut microbiota (Hoban et al., 2016b). Using GF mice, Desbonnet et al. (2014) showed that the microbiota is crucial for the development of normal social behaviors, including social motivation and preference for social novelty, while also being an important regulator of repetitive behaviors (Arentsen et al., 2015; Buffington et al., 2016). This decreased sociability has also been demonstrated in rats (Crumeyrolle-Arias et al., 2014). Interestingly, the peptidoglycan (PGN)-sensing molecule, Pglyrp2, has been shown to modulate the development of social behavior in mice and alterations in the expression of the ASD risk gene c-Met (Arentsen et al., 2017).

Oxytocin, a neuropeptide produced in the paraventricular nucleus (PVN) of the hypothalamus, is important for sociability (Teng et al., 2013). Offspring of mothers fed a high-fat diet showed reduced levels of oxytocin PVN neurons, in addition to behavioral and gut microbiota alterations (Buffington et al., 2016). *L. reuteri* treatment restored oxytocin levels and social behaviors. A recent study, using low dose penicillin, administered to dams in late pregnancy and early post-natal life showed that this antibiotic induced gut microbiota alterations, increased cytokine expression in frontal cortex, modified BBB integrity and decreased anxiety-like and social behaviors, in offspring (Leclercq et al., 2017). Interestingly, concurrent supplementation with *L. rhamnosus* (JB-1) attenuated the penicillin induced decrease in social novelty.

The maternal immune activation (MIA) model serves as a useful model for neurodevelopmental disorders such as ASD and schizophrenia, and it is well established that prenatal infection can act as “neurodevelopmental disease primer,” the consequences of which are dependent on precise timing of MIA (Meyer et al., 2006; Smith et al., 2007; Knuesel et al., 2014; Meyer, 2014; Coiro et al., 2015; Meehan et al., 2016; Pendyala et al., 2017). MIA rodents display all three of the core features of human ASD, including limited social interactions, a tendency toward repetitive behavior and reduced communication (Patterson, 2011). A recent study showed that MIA induces dysregulation of fetal brain transcriptome by downregulating genes related to ASD (Lombardo et al., 2017). MIA has been associated with altered gut microbiota. Furthermore, the commensal *Bacteroides fragilis* reversed the deficits in communicative, stereotypic, anxiety-like and sensorimotor behaviors (Hsiao et al., 2013).

Autistic like behavior and neurochemical alterations have also been demonstrated in a mouse model of food allergy (de Theije et al., 2014b). The same author showed an altered gut microbiota profile in an autism model, using valproic acid (VPA) (de Theije et al., 2014a). Interestingly, VPA, a medication used as a mood stabilizer in bipolar affective disorder and as an antiepileptic, functions as a HDAC inhibitor and has a similar structure to the SCFA propionic acid. It is well established that VPA acid use during pregnancy increases the risk of autism (Jacob et al., 2013), and propionic acid can also modulate mitochondrial function in autism and control cell lines (Frye et al., 2016).

As indicated above, multiple cognitive domains are impacted in ASD and schizophrenia and the gut microbiota has been implicated in a number of relevant cognitive functions. The combination of acute stress and infection can impact cognition. *Citrobacter rodentium* infected C57BL/6 mice that were exposed to acute stress exhibited memory dysfunction (Gareau et al., 2011). Moreover, GF Swiss-Webster mice displayed memory impairment at baseline, in the absence of acute stress (Gareau et al., 2011). In male C57BL/6 mice, higher percentages of *Clostridiale*s and lower levels of *Bacteroidales* in high-energy diets were related to poorer cognitive flexibility (Magnusson et al., 2015). In BALB/c mice, treatment with *B. Longum* resulted in an improvement in stress related behavior and cognition (Savignac et al., 2015). Hippocampal neurogenesis, a pivotal process in learning and memory consolidation (Deng et al., 2010; Levone et al., 2015; Anacker and Hen, 2017; Hueston et al., 2017) has been shown to be regulated by the gut microbiota. GF mice exhibit increased adult hippocampal neurogenesis in the dorsal hippocampus, and post-weaning microbial colonization failed to reverse the changes in adult hippocampal neurogenesis (Ogbonnaya et al., 2015). Furthermore, exercise or probiotics were able to ameliorate deficits in neurogenesis and behavior in antibiotic-treated mice (Mohle et al., 2016). A recent study showed that *L. johnsonii* CJLJ103 attenuated colitis and memory impairment in mice by inhibiting gut microbiota lipopolysaccharide production and NF-κB activation (Lim et al., 2017).

Using an antibiotic (ampicillin, metronidazole, vancomycin, ciprofloxacin, imipenem) treated rat model, gut microbiota depletion during adulthood resulted in deficits in spatial memory as measured by Morris water maze (Hoban et al., 2016a). In another pre-clinical study, that used ampicillin, bacitracin, meropenem, neomycin, and vancomycin, novel object recognition, but not spatial memory, was impaired in antibiotic-treated mice and this cognitive deficit was associated with brain region-specific changes in the expression of cognition-relevant signaling molecules, notably BDNF, N-methyl-D-aspartate receptor subunit 2B, serotonin transporter and neuropeptide Y system. The authors concluded that circulating metabolites and the cerebral neuropeptide Y system play an important role in the cognitive impairment and dysregulation of cerebral signaling molecules due to antibiotic-induced gut alterations (Frohlich et al., 2016). Furthermore, in a pre-clinical rodent model of diabetes, *L. acidophilus, B. lactis*, and *L. fermentum*, improved diabetes-induced impairment of cognitive function in the Morris water maze and synaptic activity in rats (Davari et al., 2013). The N-methyl-D-aspartate (NMDA) receptor antagonist, phencyclidine causes hyperlocomotion, social withdrawal, and cognitive impairment in rodents, and serves as a useful pharmacological rodent model of schizophrenia (Jones et al., 2011). A study investigating the effect of subchronic phencyclidine (subPCP) treatment on cognition and gut microbiota, found that the microbiota altered immediately after subPCP washout. Administration of ampicillin abolished the subPCP-induced memory deficit (Pyndt Jorgensen et al., 2015).

### Microbiota and stress

The brain interprets perceived stressors to determine physiological and behavioral responses. This process can promote adaptation (allostasis), but when responses are exaggerated or overused (allostatic overload), pathology can ensue (McEwen, 2017). The immune system and HPA axis are pivotal to the stress response and act as mediators to alter neural circuitry and function, particularly in the hippocampus, amygdala, and prefrontal cortex (McEwen et al., 2016). Stressful life events can precipitate psychotic symptoms (Day et al., 1987), and increased sensitivity to minor stressful events are associated with more intense psychotic experiences in first episode psychosis (FEP) (Reininghaus et al., 2016b). In addition early life event stressors, such as childhood trauma (Varese et al., 2012) and social adversity/defeat stressors, such as migration/ethnic minority status can increase the risk of psychosis (Elizabeth Cantor-Graae and Jean-Paul Selten, 2005; Selten and Cantor-Graae, 2005; Fusar-Poli et al., 2017). As mentioned above, schizophrenia is a highly heterogenous disorder, and commonly co-morbid with anxiety and depressive disorders (Buckley et al., 2009; Achim et al., 2011). Similarly, approximately 40% of young people with ASD have at least one comorbid DSM-IV anxiety disorder (van Steensel et al., 2011) and there are higher levels of depression (Ghaziuddin et al., 2002; Magnuson and Constantino, 2011; Strang et al., 2012).

Stress can reshape gut microbiota composition (Wang and Wu, 2005; O'Mahony et al., 2009; Galley et al., 2014a,b; Golubeva et al., 2015; Frohlich et al., 2016). For example, early life maternal separation resulted in a significant decrease in fecal *Lactobacillus* numbers on day 3 post-separation which was correlated with stress related behaviors in rhesus monkeys (Bailey and Coe, 1999). In a mouse model of social disruption, stress altered the gut microbial profile and increased the levels of the pro-inflammatory cytokine IL-6 (Bailey et al., 2011). Interestingly, it was possible to transfer an anxious behavioral phenotype between two strains of mice via fecal microbiota transfer (Bercik et al., 2011). More recently, it has been shown that mice that received an obesity associated microbiota exhibit more anxiety-like behaviors associated with increased evidence of neuroinflammation compared to controls (Bruce-Keller et al., 2015).

As previously mentioned, the developmental trajectory of the gut microbiota is compatible with concepts of the early-life period as a vulnerable phase for the subsequent emergence of neurodevelopmental disorders (O'Mahony et al., 2009, 2017; Borre et al., 2014). Pre-clinical evidence suggests that the gut microbiota signature acquired and maintained during these pivotal stages may also affect stress reactivity. GF rodents demonstrate abnormal behavioral and neuroendocrine responses to stress (Sudo et al., 2004; Nishino et al., 2013; Crumeyrolle-Arias et al., 2014; Moloney et al., 2014) and the normal development of the HPA axis is contingent on microbiota colonization at specific neurodevelopmental time points (Sudo et al., 2004).

Furthermore, the expression of anxiety like behavior in a mouse model of early life stress is partially dependent on the gut microbiota (De Palma et al., 2015). Evidence suggests that prenatal stress also impacts the gut microbiota with implications for physiological outcomes in the offspring (Golubeva et al., 2015). In a mouse model of prenatal stress, maternal stress decreased the abundance of vaginal *Lactobacillus*, resulting in decreased transmission of this bacterium to offspring, which corresponded with changes in metabolite profiles involved in energy balance, and with disruptions of amino acid profiles in the developing brain (Jasarevic et al., 2015). Human infants of mothers with high self-reported stress and high salivary cortisol concentrations during pregnancy had significantly higher relative abundances of *Proteobacterial* groups known to contain pathogens and lower relative abundances of lactic acid bacteria (*Lactobacillus*) and *Bifidobacteria* (Zijlmans et al., 2015). In addition, those infants with altered microbiota composition exhibited a higher level of maternally reported infant GI symptoms and allergic reactions, highlighting the functional consequences of aberrant colonization patterns.

The stress related disorder, depression, commonly co-morbid with ASD and schizophrenia, has been associated with alterations in gut microbiota profiles (Naseribafrouei et al., 2014; Jiang et al., 2015) and altered metabolomic output (Yu et al., 2017). Fecal Microbiota Transfer (FMT) from depressed patients to GF mice (Zheng et al., 2016) and antibiotic treated rats (Kelly, 2016; Kelly et al., 2016a) resulted in the transfer of certain depressive and anxiety like behaviors in the recipient rodents. The first study investigating the gut microbiota in bipolar affective disorder patients (*n* = 115), showed levels of *Faecalibacterium* were decreased, after adjusting for age, sex, and BMI, compared to healthy control subjects (*n* = 64). Moreover, *Faecalibacterium* was associated with better self-reported health outcomes based on the Short Form Health Survey, the Patient Health Questionnaire, the Pittsburg Sleep Quality Index, the Generalized Anxiety Disorder scale, and the Altman Mania Rating Scale (Evans et al., 2017). Interestingly, reduced levels of *Faecalibacterium* were reported in the study by Jiang and colleagues, which negatively correlated with severity of depressive symptoms (Jiang et al., 2015).

Fecal microbiota signatures in patients with diarrhea-predominant Irritable Bowel Syndrome (IBS), a stress related GI disorder, were shown to be similar to those patients with depression (Liu et al., 2016). Moreover, FMT from IBS patients to rats, induced anxiety related behaviors in the rats (De Palma and Lynch, 2017). In a double blind RCT of IBS patients, 6 weeks of *B. longum* NCC3001 reduced depression scores as measured by the Hospital Anxiety and Depression scale, and responses to negative emotional stimuli in amygdala and fronto-limbic regions, using fMRI, compared to placebo (Pinto-Sanchez et al., 2017). A recent study, using structural MRI, showed that gut microbial composition correlated with sensory and salience-related brain regions (Labus et al., 2017).

### Translational approaches

In pre-clinical studies, both prebiotic (Burokas et al., in press) and probiotic treatment can reduce stress related behaviors (Abildgaard et al., 2017; Moya-Pérez et al., 2017). In a recent study, *L. reuteri* was reported to reduce despair like behavior in mice by inhibition of intestinal Indoleamine 2,3-dioxygenase (IDO1) and decrease peripheral levels of kynurenine (Marin et al., 2017). The profusion of pre-clinical data indicating a role for the brain-gut-microbiota axis in brain development, function and behavior, prompted the growing need to translate these findings into human populations (Kelly et al., 2015). “Psychobiotics,” originally defined as live bacteria that when ingested in adequate amounts could produce a positive mental health benefit, in terms of anxiety, mood and cognition (Dinan et al., 2013), has more recently been expanded to encompass “any substance that exerts a microbiome-mediated psychological effect” (Sarkar et al., 2016; Allen et al., 2017).

The process of translating psychobiotics from bench to bedside is not without significant challenges (Arrieta et al., 2016; Kelly, 2016; Kelly et al., 2016b; Cani, 2017), but a growing number of small studies with healthy individuals suggest that prolonged pre- and probiotic consumption can positively affect aspects of mood and anxiety in healthy controls (Messaoudi et al., 2011; Mohammadi et al., 2015; Steenbergen et al., 2015; Allen et al., 2016) and modulate HPA axis function (Messaoudi et al., 2011; Schmidt et al., 2015; Allen et al., 2016). Importantly, a fermented milk containing *B. animalis, Streptococcus thermophiles, L. bulgaricus, and L. lactis*, administered for 4 weeks to healthy women, reduced the task-related response of a distributed functional network containing affective, viscerosensory and somatosensory cortices, independent of self-reposted GI symptoms (Tillisch et al., 2013).

In humans, studies investigating the potential cognitive enhancing effects of microbial based therapies are starting to emerge (Allen et al., 2016). In this study, 4 weeks of treatment with the probiotic *B. longum* 1714 modestly improved performance in a hippocampal dependent memory task in healthy volunteers. However, this effect is likely strain specific since this subtle cognitive enhancing effect was not evident following administration of *L. rhamnosus* (JB-1) (Kelly et al., 2017a). In a randomized, double-blind, placebo-controlled trial involving healthy human participants (*n* = 76), the tetracycline antibiotic doxycycline (200 mg), a matrix metalloproteinase inhibitor, resulted in reduced fear memory retention, measured with fear-potentiated startle, 7 days post-acquisition compared to participants that received placebo (Bach et al., 2017). Doxycycline can alter the composition of the gut microbiota and its metabolomic output (Angelakis et al., 2014; Behr et al., 2017). Considering the recent pre-clinical data suggesting a role for the gut microbiota in the behavioral response during amygdala-dependent memory retention (Hoban et al., 2017), it would be a compelling prospect to ascertain if alterations in gut microbiota played a physiological role in this antibiotic human study. A cross sectional MRI study comparing 20 obese individuals to 19 age and sex matched non-obese controls, reported that the relative abundance of *Actinobacteria* phylum was associated with magnetic diffusion tensor imaging variables in the thalamus, hypothalamus, and amygdala and also with to cognitive test scores related to speed, attention, and cognitive flexibility (Fernandez-Real et al., 2015). Although preliminary, these studies, and others (Pinto-Sanchez et al., 2017), are beginning to merge microbiome research with neuroimaging to further delineate the role of the gut microbiota on cognition and neural circuitry.

To date, there are two interventional studies investigating potential psychobiotics in clinical depression, with conflicting results. In the first study, 8 weeks of a multispecies probiotic containing *L. acidophilus, L. casei, and B. bifidum*, added to an SSRI, reportedly reduced depressive symptoms in moderately depressed patients compared to placebo (Akkasheh et al., 2016). The other study, conducted in antidepressant free depressed subjects, failed to show superiority of *L. helveticus* and *B. longum* over placebo, in an 8-week double blind randomized controlled trial (Romijn et al., 2017). A Mediterranean diet, suggested as protective for depression, has been associated with beneficial microbiome-related metabolomic profiles (De Filippis et al., 2015) and there is increasing awareness of the role of a healthy diet in reducing the risk of depression (Jacka et al., 2010, 2017; Opie et al., 2017). Collectively, these studies suggest that the gut microbiota may play a pathophysiological role in stress-related disorders. However, given the small sample sizes and lack of a standardized approach in these studies, a robust and consistent gut microbiota signature in stress-related disorders, remains elusive. Moreover, a systematic review found very limited evidence for the efficacy of psychobiotics in psychological outcomes (Romijn and Rucklidge, 2015). Similarly, even in GI disorders, gut microbiota analysis as a diagnostic or prognostic tool has not transitioned into routine clinical practice (Quigley, 2017).

There has been one clinical interventional study investigating probiotics in patients diagnosed with schizophrenia. This randomized, double-blind, placebo-controlled trial (*n* = 65), used *Lactobacillus rhamnosus* strain GG and *Bifidobacterium animalis* subsp. lactis strain Bb12, improved GI symptoms, but failed to impact positive or negative symptoms (Dickerson et al., 2014). A number of small studies have shown that the antibiotic minocycline, notwithstanding a complex mechanism of action, is known to modulate the brain-gut-microbiota axis, (Wong et al., 2016), and may improve negative and cognitive symptoms in schizophrenia (Miyaoka et al., 2008; Levkovitz et al., 2010; Jhamnani et al., 2013; Khodaie-Ardakani et al., 2014). This raises the question of whether microbiome based therapies could play a role in the amelioration of cognitive or negative symptoms in subgroups of psychosis spectrum disorder.

### Schizophrenia spectrum disorder and stratified psychiatry

The full neuropsychiatric implications of specific aberrations in the gut microbiota at early developmental stages or during adolescence have not been fully explored. It is an intriguing prospect that these aberrations may serve as additional risk factors or mediators for the development of psychotic disorders. It remains an unanswered question whether the gut microbiota is a state or trait marker and whether it plays a role, in conjunction with for example, stress, as a trigger factor for a psychotic relapse. The role of psychobiotics in schizophrenia remains under investigated (see Table 2). It would be interesting to explore whether a microbial based therapy could be a useful preventative strategy, or as an adjunctive agent in subgroups or whether it could reduce conversion to psychosis in subgroups at risk of developing the disorder. Well powered, longitudinal studies, encompassing neuroimaging markers would be required to definitively answer these questions.

In recent years, the categorical diagnostic system in clinical psychiatry has been challenged. Even the term schizophrenia has been disputed (van Os, 2016), with evidence showing that renaming the disorder can reduce stigma and benefit communication between clinicians, patients and families (George and Klijn, 2013; Lasalvia et al., 2015). There is growing momentum toward a more precise, dimensional approach, designed to uncover the biological mechanisms of these complex disorders. Functional dimensional constructs grouped into domains such as negative valance (acute threat (fear), potential threat (anxiety), sustained threat, loss, frustrative non-reward), positive valence (approach motivation, initial responsiveness to reward attainment, sustained/longer term responsiveness to reward attainment, reward learning, habit), cognitive (attention, perception, declarative memory, language, cognitive control, working memory), social processing (affiliation and attachment, social communication, perception, and understanding of self/others), and arousal/regulatory systems (arousal, circadian rhythms, sleep-wakefulness) examined across units of analysis from genes, molecules, cells, circuits, physiology, neuroimaging, behavior and self-report have been proposed (Insel et al., 2010). This dimensional approach is more difficult in disorders such as psychosis, compared to mood disorders, but this exciting process has begun (Reininghaus et al., 2016a; Cohen et al., 2017; Joyce et al., 2017). By deconstructing heterogenous systems-disorders (Öngür, 2017; Silbersweig and Loscalzo, 2017), such as schizophrenia into transdiagnostic constructs, and stratifying subgroups of patients based on similar pathophysiology, such as microbiome alterations and related signaling pathways, this opens up the possibility to advance personalized and precision treatments options (Kaiser and Feng, 2015).

Additionally, by removing the constraints of classical psychiatric disease diagnosis it has the potential to better align pre-clinical and clinical studies to build a common framework of comparable neurobiological abnormalities. Clearly, it is impossible to fully mimic a complex neuropsychiatric disorder such as schizophrenia or ASD in non-human animals. Hallucinations, delusions, thought disorder, and language impairments cannot be modeled. Thus, rather than modeling an entire disorder, the focus should be aimed at more precise constructs such those mentioned above, with the addition of the gut microbiota. Most currently used behavioral models do not include the gut microbiota as a factor. Clearly demonstrating causality in microbiome research is challenging (Hanage, 2014; Cani, 2017). The humanized FMT model is an integral component to demonstrate cause and effect in gut microbiota studies involving neurodevelopmental disorders such as schizophrenia, and provided a reliable and reproducible model can be developed, the precise temporal dynamics of the emergence and possible persistence of the behavioral alterations post FMT could be further delineated.

Furthermore, whether different human donor symptom profiles can be transferred via FMT may further disentangle the contribution of the gut microbiota to the pathophysiology of aspects of psychosis, by attempting to transfer sub-categories of psychotic subjects, including medication free subjects, with different constructs such as negative valance, positive valence, cognitive, social processing and arousal/regulatory systems. While it must be acknowledged that significant neuroscientific advances have frequently been lost in translation and not had appreciable benefits for psychiatric patients as yet, an evolving dimensional framework, consolidating multiple disciplines, and encompassing the gut microbiota as an additional environmental construct linked to other constructs, offers potential to identify sub-groups of patients that may be more likely to respond to a microbiome-based therapeutic approach at specific neurodevelopmental time points (Kelly, 2016; Severance et al., 2016c; Kelly et al., 2017b).

## Conclusions and perspectives

Highly complex neurodevelopmental disorders such as ASD and schizophrenia require a systems level approach. The human brain develops and functions within the context of a complex network of lifelong microbial signaling pathways from gut to brain. Pre-clinical studies are beginning to provide mechanistic insights into these signaling pathways as they relate to the social, emotional and cognitive domains of the brain. Furthermore, they suggest that psychobiotics can ameliorate certain defects. However, translating these promising pre-clinical benefits to human neurodevelopment disorders is challenging. The majority of clinical studies investigating the gut microbiota in ASD are cross sectional and underpowered, and there is insufficient evidence of solid clinical relevance. In schizophrenia, there is emerging preliminary evidence of an altered gut microbiota. An intriguing prospect would be to focus on different neurodevelopmental time points, for example during adolescence, in subgroups at risk of developing neuropsychiatric symptoms, and to encompass a dimensional construct approach. Larger prospective interventional clinical studies, with central markers of brain function, utilizing therapeutic modulation of the gut microbiota or its metabolites are required. Furthermore, exploration of the interaction of the gut microbiota and nutritional modification, at different neurodevelopmental stages, including pre-conception, warrants exploration as a preventative strategy for neurodevelopmental disorders in addition to stress-related disorders (Jacka, 2017). Although it is premature to draw firm conclusions about the clinical utility of microbiome based treatment strategies in neurodevelopmental disorders at this point, it is an exciting frontier in psychiatry research.

## Author contributions

JK wrote the manuscript. CM created the figures. GC, JC, and TD critiqued and edited the manuscript drafts.

### Conflict of interest statement

The authors declare that the research was conducted in the absence of any commercial or financial relationships that could be construed as a potential conflict of interest.
